# Feeding and breeding aspects of *Stomoxys calcitrans* (Diptera: Muscidae) under laboratory conditions

**DOI:** 10.1051/parasite/2012194309

**Published:** 2012-11-15

**Authors:** A. Salem, M. Franc, P. Jacquiet, E. Bouhsira, E. Liénard

**Affiliations:** 1 Laboratoire de Parasitologie, Université de Toulouse, INP, ENVT 31076 Toulouse France

**Keywords:** *Stomoxys calcitrans*, stable fly, rearing, blood, sugar, survivorship, *Stomoxys calcitrans*, mouche des étables, élevage, sang, sucre, survie

## Abstract

Bionomic aspects of *Stomoxys calcitrans* (Linnaeus, 1758) (Diptera: Muscidae) were studied under laboratory conditions. For this reason, laboratory-rearing techniques were optimized at the National Veterinary School of Toulouse. The colony was maintained at 25 ± 2 °C, 50 ± 10% RH under a 12-hour light cycle and observed daily. The size of each adult cage is 30 x 30 x 30 cm and designed to house about 500-1,000 flies. The average cycle from egg to adult was 19.2 ± 1.7 days. The mean longevity of imagos was 9.3 ± 5.8 days and not significantly different between sexes. Stable flies were split into two groups; the first was fed with blood, honey and water, and the second was fed only with honey and water. The mean weight of a blood meal was 11.1 ± 3.8 mg with no significant differences between males and females. The mean longevity of non-blood fed flies was found to be significantly higher (10.4 ± 3.9 days) than those fed with blood. The maximum lifespan was shorter for non-blood fed males (17 days) and females (18 days) than for those fed with blood (females: 24 days, males: 23 days). Under these laboratory conditions, *S. calcitrans* rearing was successfully established. In the end, the number of expected generations of *S. calcitrans* and the net reproduction rate were estimated to be 11.8 generations/year and 16.2 living females per female respectively.

## Abstract (fr)

Les aspects bionomiques de *Stomoxys calcitrans* (Linné, 1758) (Diptera : Muscidae) ont été étudiés en laboratoire à l’École Nationale Vétérinaire de Toulouse. Une colonie de *S. calcitrans* a été élevée dans des cages de 30 x 30 x 30 cm contenant entre 500 et 1 000 adultes par cage, à une température de 25 ± 2 °C, une humidité relative de 50 ± 10 % et avec une alternance de 12 heures d’éclairage et 12 heures d’obscurité. Dans ces conditions, la durée du cycle évolutif de l’oeuf à l’adulte a été de 19,2 ± 1,7 jours en moyenne. La longévité moyenne des imagos a été de 9,3 ± 5,8 jours et n’était pas significativement différente entre les deux sexes. Les mouches adultes ont été divisées en deux groupes après l’émergence : le premier a été alimenté avec du sang, du miel et de l’eau, pendant que le second a été nourri exclusivement avec du miel et de l’eau. Le poids moyen du repas de sang a été de 11,1 ± 3,8 mg sans différence significative entre mâles et femelles. La longévité moyenne a été significativement plus élevée (10,4 ± 3,9 jours) dans le second groupe. Toutefois, l’espérance de vie maximale était plus courte pour les mâles (17 jours) et les femelles (18 jours) sans repas sanguin en comparaison du premier groupe (mâles : 23 jours, femelles : 24 jours). Le nombre attendu de générations annuelles de *S. calcitrans* a été estimé à 11,8 générations et le taux de reproduction à 16,2 femelles vivantes par femelle.

## Introduction

The blood-feeding cosmopolitan stable fly, *Stomoxys calcitrans* (Linnaeus, 1758), is a major pest of cattle and horses. Berry *et al.* (1983) calculated that the average daily and seasonal weight losses due to the stable fly were 0.02 and 2.3 kg/animal, respectively. A ratio of one fly per animal was enough to reduce milk production by 0.7 % (Bruce & Decker, 1958; Rodriguez et al., 2005). Eventually, the economic loss for the U.S. cattle industry is estimated to reach $ 2,211 million per year (Taylor *et al.*, 2012). Adults of both sexes are blood-feeders, inflicting painful bites with their long piercing proboscis, exacerbated by the absence of anesthetic components in the saliva(Cortinas & Jones, 2006). Preferred biting sites are the lower legs of horses and cattle, the ears of dogs and the ankles of humans, sometimes leading to the development of necrotic dermatitis (Geden & Hogsette, 1994; Yeruham & Braverman, 1995). *S. calcitrans* have also been shown to transmit various pathogens such as *Anaplasma marginale* (Potgieter *et al.*, 1981), West Nile Virus (Doyle *et al.*, 2011), Enzootic Bovine Leukosis Virus after experimental infection (Weber *et al.*, 1988), and *Bacillus anthracis* (Turell & Knudson, 1987). The female human bot fly, *Dermatobia hominis*, lays her eggs on the thorax and abdomen of *S. calcitrans*. Larvae of *D. hominis* hatch and penetrate the skin of the warm-blooded host while being bitten by the stable fly (Rodríguez-Batista & Leite, 1997). *S. calcitrans* is also one of the intermediate hosts of *Habronema muscae* and *H. microstoma* (Hogsette & Farkas, 2000; Rodríguez-Batista *et al.*, 2005).

The immature stages of the stable fly develop in any wet, decaying organic matter, preferentially in animal waste mixed with soil, straw, hay or silage where females can lay their eggs (Broce *et al.*, 2005; Meyer & Petersen, 1983; Hogsette *et al.*, 1987). There are many reports indicating successful completion of the entire life cycle of *S. calcitrans* under laboratory conditions (see for example Schoof, 1964 and Christmas, 1970). Sutherland (1980), Lysyk (1998) and Gilles *et al.* (2005a, b; 2007) described in depth the effects of temperature on adult longevity and life cycle chronology. The objective of this project was to develop a simple and repeatable method for laboratory breeding of *S. calcitrans* adapted from previous works (Schoof, 1964; Christmas, 1970; Lysyk, 1998; Gilles *et al.*, 2005a, b). The rearing yield parameters, the life cycle timing and dietary characteristics of the adult fly as well as the consequences of the absence of blood from the diet on longevity and fertility, were recorded in order to assess the impact of our laboratory conditions on stable fly biology.

## Materials and Methods

### *Stomoxys Calcitrans* Rearing

#### • Colony origins

A colony of *S. calcitrans* was established in the Laboratory of Parasitology at the Veterinary National School of Toulouse (E.N.V.T.). Adult stable flies were manually removed from cattle or caught by using six Vavoua traps for 12 hours in May 2009 at the campus of the E.N.V.T. This trap was initially developed in West Africa for the control of tsetse flies (Laveissière & Grébaut, 1990), but is now also used to capture *S. calcitrans* (Gilles *et al.*, 2007; Liénard *et al.*, 2011). *S. calcitrans* were able to be identified according to Zumpt (1973). Each stable fly was examined individually under a magnifying glass to discard individuals with phoretically-attached macrochelid mites. These mites are predators of eggs and larvae (Beresford & Sutcliffe, 2009). Eventually, 53 adult stable flies formed a group of 29 males and 24 females. Christmas (1970) and Berkebile *et al.* (2009) reared immature and adult stable flies at 23 ± 2 °C, 30-50 % relative humidity (RH), under a 12 Light:12 Dark (L:D) photoperiod. Better results were obtained by Lysyk (1998) at 25 °C. Thus, based on these previous data, the stable fly colonies at the E.N.V.T. and the various experimentations were held in a dedicated room at 25 ± 2 °C, 50 ± 10 % RH and under a 12L:12D photoperiod. Humidity and temperature are checked daily and adjusted if necessary.

#### • Adult fly cage and feeding

Adult stable flies were maintained in mesh cages (30 × 30 × 30 cm), with a small circular door measuring15 cm in diameter. Each cage was suited to hold 500 to 1,000 adult flies. Adult flies were fed daily with a double-chambered glass feeder made by a local glassblower. The water was heated (38.5 °C) with an electric heat pump (ED-5 Heating Circulator with Open Bath, Julabo, Seelbach, Germany) and circulated through the outer chamber. The inner chamber containing bovine blood was sealed with a thin synthetic membrane (Parafilm 3M, Pechiney Plastic Packaging, Chicago, IL), which was placed in contact with the upper side of the cage. Stable flies fed by piercing the membrane through the mesh of the cage. Blood was collected weekly in 4-mL tubes containing 60 USP U Lithium Heparin (Terumo Europe N.V., Leuven, Belgium) to prevent coagulation (Berkebile *et al.*, 2009). Blood was stored at 4 °C for up to a week. The blood was provided by a 14-month-old calf reared at the E.N.V.T. that was not treated against flies with topical or systemic insecticides within three months before the start of the study. The 8-mL heparinized blood meal and the membrane were changed daily to prevent microbial development and blood depletion. Moreover, a cotton ball of 3 g soaked in 30 mL of tap water and 1 mL of liquid honey (Miel Mille Fleurs, La Générale des Miels, Château-Renard, France) were also changed and provided daily.

#### • Egg collection

A 40 × 50 × 15 cm plastic receptacle containing a 2- to 3-cm layer of 100 g of vermiculite (Vermex M, Efisol, Nanterre, France) and a 2- to 3-cm grass layer saturated with 300 mL of water was prepared to induce oviposition. Fresh green grass including a homogeneous blend of one-third of meadow fescue (*Festuca pratensis* Huds.), one-third of cocksfoot (*Dactilis glomerata* L.) and one-third of ryegrass (*Lolium perenne* L.), was collected from the fields around the E.N.V.T. Sterilization of 100 g of grass was performed at high temperature (250 °C for 30 minutes). The grass was left for fermentation at room temperature for six days before use. Grass and vermiculite layers were dampened with 300 mL of water. A thin piece of black cloth (30 × 30 cm) was moistened and placed between the mesh bottom of the fly cages and the oviposition medium. The moisture content of the oviposition medium was maintained by the daily addition of 50 ml of water. Eggs were collected from the black cloth by rinsing it with fresh water using a pipette and transferred to the larval rearing pan.

#### • Larval rearing

The vermiculite provided an adequate ventilated environment for *S. calcitrans* larvae (Goodhue & Cantrell, 1958; Schoof, 1964). It also allowed for free larval movement and facilitated the removal of pupae from the medium. The larval medium (100 g of vermiculite and 160 g of larval food mixture) was placed in 30 × 40 × 10 cm pans. The pan was tilted 30° to which 300 mL of water was added to dampen the substrate through capillar action. The larval food mixture was initially prepared with 125 g of crushed dry dog food (23 % protein, 16 % fat, 6.6 % ash, and 1.4 % fiber, Royal Canin Medium Adult, Royal Canin, Aimargues, France), 10 g of beer yeast (*Saccharomyces* sp.) and 25 g of blood meal (Sang desséché, Solabiol, Marseille, France), manually mixed with vermiculite. A piece of gauze covered the basin to prevent larvae from escaping. Then, 25 g of vermiculite with 15 g of the larval food mixture were daily sprinkled on top of the existing medium. Every day, 100 mL of tap water were poured into the bottom of the pan.

#### • Pupation and adult emergence

Every 24 hours, the larval rearing medium was stirred with a spatula and pupae were manually collected. Pupae were kept daily in 12 × 8 × 5 cm plastic flasks, each containing 5 g of vermiculite and 15 mL of water, covered with a piece of gauze. After emergence, imagos were transferred to the adult cage as previously described.

### Rearing Yield Parameters

#### • Timing and cycle productivity

To assess the life cycle in our laboratory conditions, 100 newly laid eggs were collected. Developmental stages were monitored daily and the survival of eggs, pupae and adults were recorded to assess the overall breeding performance. The experiments were continued until the death of all stable flies of the cohort. The test was replicated ten times.

#### • Characteristics of blood meal

Characteristics of the first meal: just after the emergence of imagos, a first group of 60 stable flies (30 males and 30 females) was isolated. Each fly was individually put in 7 × 4 cm flasks, which were sealed with netting. Blood was given on a one-time basis as the only food source to this cohort. Blood intake for the first meal was recorded by evaluating the fly’s weight difference before and after the blood meal, and the duration of the meal. Characteristics of the different meals during lifetime: a second group of 60 stable flies (30 males and 30 females) was followed until the death of all individuals. Blood was given twice a day: the first time between 8:00 and 10:00 a.m. and the second time between 16:00 and 18:00 p.m. Flies were weighed before and after each blood meal. The number of observed blood meals and amount of blood intake as well as adult longevity were also recorded.

#### • Longevity according to the type of diet

Two groups of 100 newly emerged stable flies were maintained in two adult cages. Each group received one type of diet *ad libitum*: one was given blood, honey and water and the other honey and water only. Treatments were replicated three times. The number of dead females and males was recorded daily and used to compute Kaplan-Meier survival curves (Kaplan & Meier, 1958).

#### • Fecundity and fertility

The parameters used to define the rearing performance included: net reproduction rate, mean generation time, intrinsic and finite rates of natural increase. Under controlled rearing conditions, 100 newly emerged stables flies (sex ratio close to 1:1) were placed in adult cages and continuously fed with blood, honey and water. The number of eggs laid in the cages was counted daily until the death of all females. Then, based on observed mortality rates from egg to imago and sex ratio at emergence, the life table was computed including these corrections to determine x (the age in days from egg hatching to adult death), X (age in days of adults from emergence), m_x_ (average number of potential adult females with a female aged X with an observed mortality rate of 0.468 and an observed sex ratio of 0.486) and l_x_ (percentage of surviving females from birth to age X). Reproductive and population parameters were defined using the net reproduction rate (*R_0_* = Σ l_x_.m_x_), mean generation time (*T* = Σ l_x_.m_x_.x / Σ l_x_.m_x_), intrinsic rate of natural increase (*r* = ln *R_0_* / *T*) and finite rate of natural increase (λ = e*^r^*). Four replicates of 100 stable flies were used.

### Statistical Analysis

The permutation test with exact inference was used to compare both sexes in terms of emergence delay, body weights, duration of blood meal, and lifespan according to the type of diet. It was also used to compare the mean weight of ingested blood between a single meal and two meals a day. The Spearman’s coefficient *r^2^* was computed to assess the correlation between the weight of ingested blood and that of *S. calcitrans* before feeding and between the duration of a blood meal and amount of blood intake. Comparisons between weights of ingested blood over time were made with the Friedman’s test. Kaplan-Meier survival curves regarding sex and diet compositions on the survivorship of *S. calcitrans* were constructed (Kaplan & Meier, 1958). Significances of differences between curves were estimated by Log-Rank tests assuming a proportional hazard model. Statistical analyses were carried out using the software package StatXact^®^ release 3.1 (CytelSoftware Corporation, USA), Microsoft^®^ Excel 2007 (Microsoft Corporation, USA) and R package release 2.14 (available at http://www. r-project.org) with Rcmdr Survival Plug-In version 1.0 (Leucuţa & Achimaș Cadariu, 2008). The significance threshold for overall analysis was 0.05.

## Results

### Rearing Yield Results

Table I shows the chronology of the life cycle of *S. calcitrans* under our laboratory conditions. The absence of heterogeneity between the ten replicates was checked and data from all replicates were pooled. The total duration of development from egg to imago was 19.2 ± 1.7 days. Table II presents percentages of successful production at each developmental stage. The survival rate from egg to adult is 46.8 ± 11.9 % with the highest mortality rates before pupation. All imago emergences occurred between seven to ten days after pupation and the mean sex ratio (males: females) is close to unity with a value of 1.1:1. Based on a final number of 467 hatching pupae after ten replicates, males emerged earlier than females, 8.1 ± 0.8 and 8.4 ± 0.8 days, respectively (*p* < 0.0001). After day 8, the sex ratio reverses in favor of females. The first observed mating occurred at 5.5 ± 1 days (from day 4 to day 7) and the first egg laying was observed 7.8 ± 0.8 days (ranging from day 7 to day 9) after emergence. Eventually, the life cycle from egg to egg under our laboratory conditions was 27 ± 1.9 days with a range of 23 to 32 days.

**Table I.** Range and mean with standard deviation (S.D.) of *Stomoxys calcitrans* developmental time in days under laboratory conditions (25 ± 2 °C, 50 ± 10 % RH, 12L:12D) based on ten replicates of 100 eggs.

	Duration (days)		
	
Life cycle stages	Range	Mean (± S.D.)
Egg	1 – 2	1.4 (0.5)
Larval instars	8 – 11	9.6 (0.9)
Pupae	7 – 10	8.2 (0.9)

From egg to adult	16 – 23	19.2 (1.7)

**Table II.** Range and mean percentage production with standard deviation (S.D.) of *Stomoxys calcitrans* pupae and adults, by total and by sex under laboratory conditions (25 ± 2 °C, 50 ± 10 % RH, 12L:12D) based on ten replicates of 100 eggs.

	Percentage		
	
Life cycle stages	Range	Mean (± S.D.)
Pupae (from egg)	26.0 – 65.0	50.2 (12.8)
Adult (from pupae)	86.2 – 100	93.3 (4.3)
Adult (from egg)	24.0 – 59.0	46.8 (11.9)

Male adult	40.5 – 64.2	51.4 (7.6)
Female adult	35.8 – 59.5	48.6 (7.6)

### Characteristics of Blood Meal

**Fig 1.**
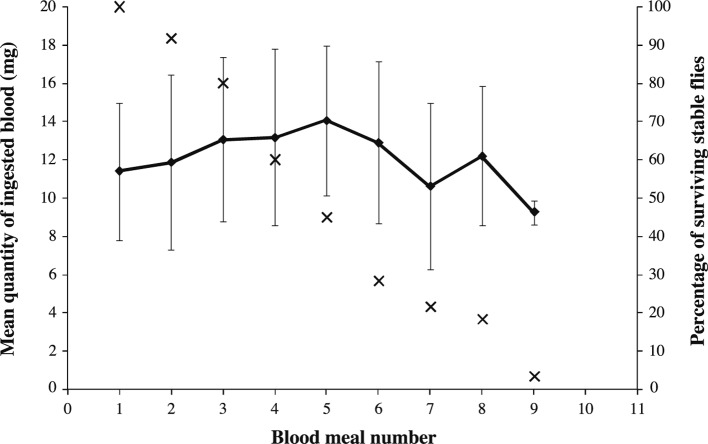
Mean quantity of ingested blood by meal and by adult stable fly with standard deviation (mg – black squares) and survivorship curve percentage of *Stomoxys calcitrans* (black crosses) according to the number of blood meal in laboratory conditions (25 ± 2 °C, 50 ± 10 % RH, 12L:12D).

The mean weights of male and female *S. calcitrans* after emergence and before the first blood meal were not significantly different (*p* = 0.212): 6.8 ± 2.3 mg (males, n = 30) and 7.5 ± 2.1 mg (females, n = 30). The mean weight of the first blood meal was 11.1 ± 3.8 mg and ranged from 3.2 to 18.7 mg. The weight of intake (y, mg) was linearly and significantly related to the stable fly’s weight before feeding (x, mg) (y = 1.405 + 1.355x, *r^2^* = 0.623, *p* < 0.0001). The mean duration of a blood meal was 126 ± 42 s and was not significantly different between males and females (*p* = 0.287). Duration was not significantly correlated with the quantity of blood ingested (*r^2^* = 0.004, *p* < 0.979). In the second group of 60 stable flies (sex ratio 1:1), the number of blood meals was taken into account for each fly until death and varied from one to nine (Fig. 1). All flies fed at least once. The median number of intakes was four and the mean delay between two meals was 1 d 7 h 33 min ± 7 h 3 min. When fed only with blood, the maximum longevity observed for stable flies was 13 days and the median survivorship was seven days for males and 6.5 days for females. The difference between both sexes was not significant (*p* = 0.472). Eight flies were fed two meals per day corresponding to a total of 16 meals. The mean weight of those 16 blood meals was 8.5 ± 3.6 mg and was significantly lower than the mean weight of the 263 single meals ingested by the other 53 stable flies (12.2 ± 4.3 mg, *p_unilateral_* < 0.0001). Only two stable flies were fed nine times (Fig. 1). A cohort of 11 stable flies that took eight meals was defined. An increase in mean quantity of ingested blood was observed from meal one to meal five. A decrease from meal five to meal seven was reported (Fig. 1). However, the difference of blood intake was not significant between meals from meal one to meal eight (*p* = 0.35). For overall meals and both virgin males and females, the mean intake was 12.4 ± 4.2 mg and ranged from 3.2 mg to 22.6 mg.

### Survival According to the Type of Diet

**Fig 2.**
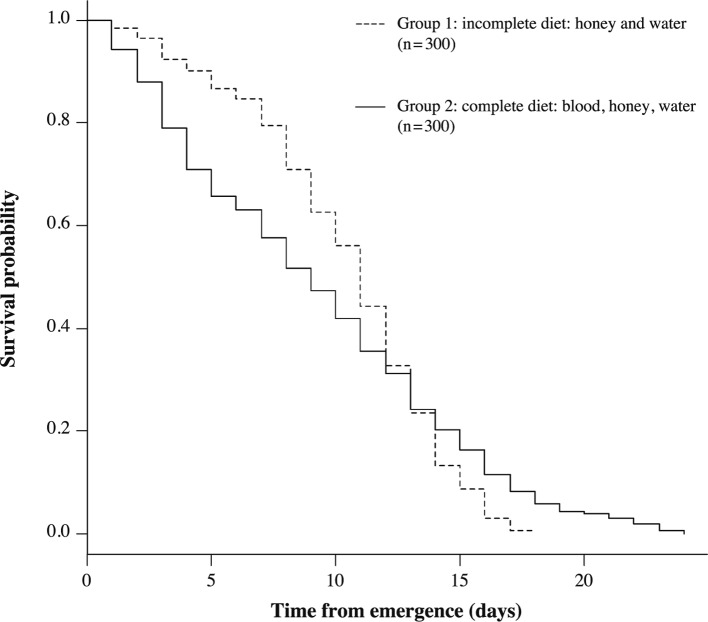
Survivorship curves of adult *Stomoxys calcitrans* fed on blood, honey and water (solid line), on honey and water only (dashed line) in laboratory conditions (25 ± 2 °C, 50 ± 10 % RH, 12L:12D).

No heterogeneity was highlighted in survival patterns between replicates within the same diet group (complete diet: blood, honey and water in one hand and honey and water only in the other hand). No significant heterogeneity was observed in male or female survival patterns within the group fed with blood, honey and water (three comparisons between three replicates within each sex, male set and female set respectively). Similarly, there was no significant difference in survivorship patterns for males or females fed only with honey and water. Statistical assumptions required for pooling data were met, thus, the replicates were pooled for further analysis. The survivorship curves between both sexes fed on a complete meal were not significantly different (χ^2^ = 3.3; *p* = 0.068). The mean longevities of female and male *S. calcitrans* were 9.9 ± 5.9 days and 8.7 ± 5.6 days, respectively, and were not significantly different (*p* = 0.083). The maximum lifespan of imagos was 24 days for females and 23 days for males under laboratory conditions. The survivorship curves between both sexes fed with honey and water only were not significantly different (χ^2^ = 0.1; *p* = 0.771). The mean lifespan of females and males was 10.4 ± 4.1 days and 10.5 ± 3.8 days, respectively, and the difference was not significant (*p* = 0.787). The maximum life expectancy of adults was shorter with this type of diet than with the blood-, honey- and water-based diet: 18 days for females and 17 days for males. Fig. 2 illustrates the two survivorship curves between both groups according to their diet. The proportional hazards assumption over time is not verified because the two curves cross. Therefore, the log-rank test cannot be computed to compare the curves. The survivorship curve of *S. calcitrans* fed with honey and water (group 1) shows that most deaths occur in later life with an increase of the hazard ratios (Fig. 2). The survivorship curve of *S. calcitrans* with a diet based on blood, honey and water (group 2) is characterized by a higher life expectancy for older individuals, after the risky juvenile period (Fig. 2). The median of group 2 (nine days) is lower than that of group 1 (11 days, *p* = 0.0006). Despite the maximum longevity of group 2, the mean lifespan of group 1 (10.4 ± 3.9 days) was significantly higher than that of group 2 (9.3 ± 5.8 days, *p* = 0.0041). No eggs were reported in group 1.

### Fecundity and Fertility

To estimate the potential yield of our colony, the life table was established (Table III). The sex ratio was maintained close to 1:1 during all four replicates with a total of 196 females, 204 males and 6,545 eggs were deposited during the oviposition period of 14 days (Table III). Data were pooled to assess the fecundity of females since no significant heterogeneities were observed between the four replicates. At the first oviposition (day 7), 146 females were alive. A decrease was observed for all replicates at day 13 between both peaks of 11 eggs/female/day (Table III). The average mortality rate from egg to adult (0.468) and the mean sex ratio (0.486) were included in the computation of mx defined in our study as the number of living adult females coming from females at pivotal age X. Using these data, the net reproduction rate (*R_0_*), the intrinsic rate of natural increase (*r*), the finite rate of natural increase (λ) and the mean generation time (*T*) of *S. calcitrans* were 16.2 living females/female, 0.09 female/female.d^-1^, 1.1 female/female.d^-1^ and 31 (30.6- 31.8) days respectively. Under laboratory conditions, the expected number of *S. calcitrans* generations was estimated to be 11.8 generations per year.

**Table III.** Life fertility table of *Stomoxys calcitrans* under laboratory conditions (25 ± 2 °C, 50 ± 10 % RH, 12L:12D) based on four replicates.

X (days)	Number of eggs/female/day	m_x_	l_x_
1	0.00	0.00	1.00
2	0.00	0.00	0.97
3	0.00	0.00	0.90
4	0.00	0.00	0.86
5	0.00	0.00	0.81
6	0.00	0.00	0.77
7	0.21	0.05	0.74
8	1.83	0.42	0.70
9	7.77	1.77	0.64
10	9.20	2.09	0.58
11	10.71	2.43	0.48
12	11.00	2.50	0.39
13	8.11	1.85	0.32
14	10.96	2.49	0.26
15	10.67	2.43	0.23
16	10.85	2.47	0.17
17	9.13	2.08	0.12
18	7.88	1.79	0.09
19	6.46	1.47	0.07
20	5.40	1.23	0.05
21	0.00	0.00	0.04
22	0.00	0.00	0.04
23	0.00	0.00	0.03
24	0.00	0.00	0.01
25	0.00	0.00	0.00

X: age of females in days since emergence; mx: average number of potential adult females by a female aged x (from birth to death including mean immature developmental time as computed in the Table I) and computed with mortality rate of 46.8 % and sex ratio of 48.6 % (Table II); l_x_: percent female survivors to age X; total number of females = 196.

## Discussion

According to previous works (Ashrafi, 1964; Christmas, 1970; Gilles *et al.*, 2005a, b; Berkebile *et al.*, 2009), our adaptations of breeding procedures have allowed the achievement of a complete life cycle and reproduction of *S. calcitrans* since May 2009. The protocol is quite simple and all stages can be reared in one room. Data on life cycle chronology and percentage of production under these laboratory conditions ranged within mean values according to Lysyk (1998) and Gilles *et al.* (2005a). High mortality occurred before pupation probably partly due to the accidental introduction of macrochelids from adult stable flies. Indeed, despite the individual examination, *Macrocheles* sp. mites completed their life cycle in our rearing conditions. They are one of the greatest predators of eggs, larvae and pupae of *S. calcitrans* (Smith *et al.*, 1989). After the collection of data for this study, the eradication of mites in the stable fly rearing was performed with amitraz-impregnated plastic strips registered for the control of *Varroa jacobsoni* Oud. 1904 in honey bees (Apivar™, Veto-Pharma, Villebon-sur-Yvette, France) in cages of adults. No mortality peak of *S. calcitrans* was observed following the introduction of amitraz. However, the impact on the stable fly’s life cycle should be assessed.

Male imagos of *S. calcitrans* emerged before females but the weights of females and males were not significantly different after emergence. Thus, a possible explanation lied in the fact that the developmental time of female larval instars and pupae were longer than those of males since more time and energy were required for the complete development of the genital tract (Aguiar-Valgode & Milward-de-Azevedo, 1992).

The mean weight of a blood meal was positively and linearly correlated to the body weight of stable flies (Dougherty *et al.*, 1995). Complete feeding was achieved in 1.4 to 2.8 min without disturbance. This duration was close to the time range of 2 to 5 min for stable flies that fed on grazing cattle (Dougherty *et al.*, 1995). The mean amount of blood intake was also close to that of a previous study (Smith & Hansens, 1975; Dougherty *et al.*, 1995). Eventually, the blood meal characteristics were not different in our laboratory conditions in comparison with a natural blood meal taken from cattle (Smith & Hansens, 1975).

The kinetics of blood meal weights was similar to those published by Venkatesh & Morrison (1980) regarding the weight of blood meals of unmated females: an increase of weight for each blood meal until the fifth meal was followed by a decrease for the next meals. The lack of mating activity could explain our observed results characterized by insignificant variations in blood intake with age. For female *S. calcitrans*, oogenesis follows the ingestion of blood but no egg laying can occur and nor can a new oocyte mature in absence of mating activity (Venkatesh & Morrison, 1980). For male *S. calcitrans*, Anderson (1978) shows that a blood meal is required for the maturation of the accessory gland essential to sperm transfer and female insemination. After each insemination, the size of the accessory gland decreased (Anderson, 1978). Then, mating activity for both sexes required additional energy inducing an increase in ingested blood (Venkatesh & Morrison, 1982).

Most stable flies ate only once a day. Some of them took two meals per day. In this case, the weight of those meals was significantly lower than that of single meals. This suggests that the first ingestion of a blood meal was insufficient to complete the daily food intake. According to these results, it may be necessary to provide fresh blood either *ad libitum* or at least twice a day in order to maintain effectively the stable fly rearing.

The mean longevities of males and females were not significantly different regardless of the diet. Those values are close to those reported by Gilles *et al.* (2005b) for blood-fed adults reared at 25 °C. The maximum longevity was observed for females fed with the complete diet (bovine blood, honey and water) without reaching 40 days as previously reported (Lysyk, 1998; Gilles *et al.*, 2005b). Those longevity variations were also reported by Lysyk (1998) between populations from Texas and South Africa. The most striking result is the discrepancy concerning the extended mean longevity for both sexes fed only with honey and water. Moreover, the maximum lifespan was considerably greater than seven days as reported by previous studies (Moobola & Cupp, 1978). Females of *Aedes albopictus* that fed on sucrose and blood did not live significantly longer than females fed only with sucrose (Xue *et al.*, 2010). Then, the relation between the survivorship and nutrition of *S. calcitrans* remains relatively blurred and requires further studies. Some points could be suggested from this study. Firstly, no high mortality rates were observed in the early life of adults as reported by Gilles *et al.* (2005b). This could be due to the high quality of our larval food which allows for the accumulation of reserves during larval instars that reached the adult stage (Gadelhak *et al.*, 1995) in comparison with elephant grass alone (Gilles *et al.*, 2005a, b). Secondly, the full completion of reproduction required energy and nutrients (Venkatesh & Morrison, 1980). No reproduction occurred without blood feeding as observed by Moobola & Cupp (1978). All larval reserves and ingested honey could be used to maintain the basal metabolism. Indeed, a sucrosebased diet did not trigger triacylglycerol synthesis as the source of energy for lipogenesis (Venkatesh & Morrison, 1980). When reserves were not available or provided by carbohydrates, the mortality increased as suggested by the shape of the survivorship curve of *S. calcitrans* fed with honey and water (Fig. 1). This curve showed an increase of mortality occurring in later life with an increase of the hazard ratios. The survivorship curve of *S. calcitrans* fed with blood, honey and water was more uniform during the adult life, probably due to the regular protein supply of the blood.

In our laboratory conditions, the lifetime egg production was 14 days which is slightly shorter than that indicated in recent reports (Lysyk, 1998; Gilles *et al.*, 2005b) but lifetime fecundity and egg production ranges are variable according to various studies (Foil & Hogsette, 1994). Interestingly, the life-history parameters of our rearing, including reproductive performances, fell in ranges of values of the net reproduction rate (*R_0_* = 16.23 living females/female), the intrinsic rate of natural increase (*r* = 0.09 female/ female.d^-1^,), the finite rate of natural increase (λ = 1.09 female/female.d^-1^) and the mean generation time (*T* = 31.04 days) computed by other authors for an optimal temperature of 25 °C (Lysyk, 1998; Gilles *et al.*, 2005a).

To conclude, entire life cycles were able to be completed in a single room with a rather simple protocol that could be adapted in various countries. Given the significant reproduction results, *S. calcitrans* may be used in further studies involving either insecticide sensitivity, which lacks dramatically for this species extended mean longevity for both sexes fed only with honey and water. Moreover, the maximum lifespan was considerably greater than seven days as reported by previous studies (Moobola & Cupp, 1978). Females of *Aedes albopictus* that fed on sucrose and blood did not live significantly longer than females fed only with sucrose (Xue *et al.*, 2010). Then, the relation between the survivorship and nutrition of *S. calcitrans* remains relatively blurred and requires further studies. Some points could be suggested from this study. Firstly, no high mortality rates were observed in the early life of adults as reported by Gilles *et al.* (2005b). This could be due to the high quality of our larval food which allows for the accumulation of reserves during larval instars that reached the adult stage (Gadelhak *et al.*, 1995) in comparison with elephant grass alone (Gilles *et al.*, 2005a, b). Secondly, the full completion of reproduction required energy and nutrients (Venkatesh & Morrison, 1980). No reproduction occurred without blood feeding as observed by Moobola & Cupp (1978). All larval reserves and ingested honey could be used to maintain the basal metabolism. Indeed, a sucrosebased diet did not trigger triacylglycerol synthesis as the source of energy for lipogenesis (Venkatesh & Morrison, 1980). When reserves were not available or provided by carbohydrates, the mortality increased as suggested by the shape of the survivorship curve of *S. calcitrans* fed with honey and water (Fig. 1). This curve showed an increase of mortality occurring in later life with an increase of the hazard ratios. The survivorship curve of *S. calcitrans* fed with blood, honey and water was more uniform during the adult life, probably due to the regular protein supply of the blood.

In our laboratory conditions, the lifetime egg production was 14 days which is slightly shorter than that indicated in recent reports (Lysyk, 1998; Gilles *et al.*, 2005b) but lifetime fecundity and egg production ranges are variable according to various studies (Foil & Hogsette, 1994). Interestingly, the life-history parameters of our rearing, including reproductive performances, fell in ranges of values of the net reproduction rate (*R0* = 16.23 living females/female), the intrinsic rate of natural increase (*r* = 0.09 female/ female.d-1,), the finite rate of natural increase (λ = 1.09 female/female.d-1) and the mean generation time (*T* = 31.04 days) computed by other authors for an optimal temperature of 25 °C (Lysyk, 1998; Gilles *et al.*, 2005a).

To conclude, entire life cycles were able to be completed in a single room with a rather simple protocol that could be adapted in various countries. Given the significant reproduction results, *S. calcitrans* may be used in further studies involving either insecticide sensitivity, which lacks dramatically for this species or vector competence for the transmission of various pathogens such as *Besnoitia besnoitia* or *Trypanosama evansi* under controlled conditions.

## References

[R1] Aguiar-Valgode M. & Milward-de-Azevedo E.M.V. Determinacao das exigências térmicas de *Stomoxys calcitrans* (L.) (Diptera, Muscidae), em condicöes de laboratório [Portuguese]. Memórias do Instituto Oswaldo Cruz, 1992, 87, 11–20 1343786 10.1590/s0074-02761992000500005

[R2] Anderson J.R. Mating behavior of *Stomoxys calcitrans*: Effects of a blood meal on the mating drive of males and its necessity as a prerequisite for proper insemination of females. Journal of Economic Entomology, 1978, 71, 379–386 649836 10.1093/jee/71.2.379

[R3] Ashrafi S.H. The cultivation and nutritional requirements of *Stomoxys calcitrans*. Bulletin of the World Health Organization, 1964, 31, 519–520 14272458 PMC2555015

[R4] Beresford D.V. & Sutcliffe J.F. The effect of *Macrocheles muscaedomesticae* and *M. subbadius* (Acarina: Macrochelidae) phoresy on the dispersal of *Stomoxys calcitrans* (Diptera: Muscidae). Systematic & Applied Acarology Special Publications, 2009, 23, 1–30

[R5] Berkebile D.R., Weinhold A.P. & Taylor D.B. A new method for collecting clean stable fly (Diptera: Muscidae) pupae of known age. Southwestern Entomologist, 2009, 34, 469–476

[R6] Berry I.L., Stage D.A. & Campbell J.B. Populations and economic impacts of stable flies on cattle. Transactions of the ASAE, 1983, 873–877

[R7] Broce A.B., Hogsette J. & Paisley S. Winter feeding sites of hay in round bales as major developmental sites of *Stomoxys calcitrans* (Diptera: Muscidae) in pastures in spring and summer. Journal of Economic Entomology, 2005, 98, 2307–2312 16539164 10.1603/0022-0493-98.6.2307

[R8] Bruce W.N. & Decker G.C. The relationship of stable fly abundance to milk production in dairy cattle. Journal of Economic Entomology, 1958, 52, 269–274

[R9] Christmas P.E. Laboratory rearing of the biting fly *Stomoxys calcitrans* (Diptera: Muscidae). New Zealand Entomologist 2009, 4, 45–49

[R10] Cortinas R. & Jones C.J. Ectoparasites of cattle and small ruminants. Veterinary Clinics Food Animal Practice, 2006, 22, 673–693 17071359 10.1016/j.cvfa.2006.06.003

[R11] Cruz-Vazquez C. Vitela M.I. Ramos P.M. & Garcia-Vazquez Z. Influence of temperature, humidity and rainfall on field population trend of *Stomoxys calcitrans* (Diptera: Muscidae) in a semiarid climate in Mexico. Parasitología latinoamericana, 2004, 59, 99–103

[R12] Dougherty C.T., Knapp F.W., Burrus P.B., Willis D.C. & Cornelius P.L. Behaviour of grazing cattle exposed to small population of stable flies (*Stomoxys calcitrans* L.). Applied Animal Behaviour Science, 1995, 42, 231–248

[R13] Doyle M.S., Swope B.N., Hogsette J.A., Burkhalter K.L., Savage H.M. & Nasc R.S. Vector competence of the stable fly (Diptera: Muscidae) for West Nile Virus. Journal of Medical Entomology, 2011, 48, 656–668 21661328 10.1603/me10167

[R14] Foil L.D. & Hogsette J.A. Biology and control of tabanids, stable flies and horn flies. Scientific and Technical Review of the Office International des Epizooties, 1994, 13, 1125–1158 10.20506/rst.13.4.821 7711307

[R15] Gadelhak G.G., Pedibhotla V.K., Rosario R.M.T., Thomas G.D. & Stanley-Samuelson D.W. The influence of blood meals on accumulation of arachidonic acid by adult stable flies. Comparative Biochemistry and Physiology, 1995, 110B, 613–621

[R16] Geden C.J. Hogsette J.A. Research and extension needs for integrated pest management for arthropods of veterinary importance. Proceedings of a Workshop in Lincoln, Nebraska, 1994, 56–64

[R17] Gilles J., David J.-F. & Duvallet G. Temperature effects on development and survival of two stable flies, *Stomoxys calcitrans* and *Stomoxys niger niger* (Diptera: Muscidae), in La Reunion Island. Journal of Medical Entomology, 2005a, 42, 260–265 15966110 10.1093/jmedent/42.3.260

[R18] Gilles J., David J.-F. & Duvallet G. Effects of temperature on the rate of increase of *Stomoxys calcitrans* and *Stomoxys niger niger* (Diptera: Muscidae) from La Reunion Island. Journal of Medical Entomology, 2005b, 42, 959–965 16465735 10.1093/jmedent/42.6.959

[R19] Gilles J., David J.-F., Duvallet G., De La Rocque S. & Tillard E. Efficiency of traps for *Stomoxys calcitrans* and *Stomoxys niger niger* on Reunion Island. Medical and Veterinary Entomology, 2007, 21, 65–69 17373948 10.1111/j.1365-2915.2006.00658.x

[R20] Goodhue L.D. & Cantrel K.E. The use of vermiculite in medium for stable fly larvae. Journal of Economic Entomology, 1958, 51, 250

[R21] Hogsette J.A. & Farkas R. Secretophagous and hematophagous higher Diptera. Contributions to a Manual of Palaearectic Diptera, 2000, 1, 769–792

[R22] Hogsette J.A., Ruff J.P. & Jones C.J. Stable fly biology and control in Northwest Florida. Journal of Agricultural Entomology, 1987, 4, 1–11

[R23] Kaplan E.L. & Meier P. Nonparametric estimation from incomplete observations. Journal of the American Statistical Association, 1958, 53, 457–481

[R24] Laveissière C. & Grébaut P. Recherche sur les pièges à glossine (Diptera : Glossinidae). Mise au point d’un modèle économique : le piège Vavoua [French]. Tropical Medicine and Parasitology, 1990, 41, 185–192 2166330

[R25] Leucuţa D.C. & Achimaș Cadariu A. Statistical graphical user interface plug-in for survival analysis in R statistical and graphics language and environment. Applied Medical Informatics, 2008, 23, 57–62

[R26] Liénard E., Salem A., Grisez C., Prévot F., Bergeaud J.P., Franc M., Gottstein B., Alzieu J.P., Lagalissed Y. & Jacquiet P. A longitudinal study of *Besnoitia besnoiti* infections and seasonal abundance of *Stomoxys calcitrans* in a dairy cattle farm of southwest France. Veterinary Parasitology, 2011, 177, 20–27 21185653 10.1016/j.vetpar.2010.11.030

[R27] Lysyk T.J. Relationships between temperature and life-history parameters of *Stomoxys calcitrans* (Diptera: Muscidae). Journal of Medical Entomology, 1998, 35, 107–119 9538570 10.1093/jmedent/35.2.107

[R28] Meyer J.A. & Petersen J.J. Characterization and seasonal distribution of breeding sites of stable flies and house flies (Diptera: Muscidae) on eastern Nebraska feedlots and dairies. Journal of Economic Entomology, 1983, 76, 103–108 6826881 10.1093/jee/76.1.103

[R29] Moobola S.M. & Cupp E.W. Ovarian development in the stable fly, *Stomoxys calcitrans*, in relation to diet and juvenile hormone control. Parasitological Entomology, 1978, 3, 317–321

[R30] Potgieter F.T., Sutherland B. & Biggs H.C. Attempts to transmit *Anaplasma marginale* with *Hippobosca rufipes* and *Stomoxys calcitrans*. Journal of Veterinary Research, 1981, 48, 119–122 7312304

[R31] Rodríguez-Batista B.Z. & Leite R.C. Ocurrence of biological vectors of *Dermatobia hominis* (L. Jr., 1781) (Diptera: Cuterebridae), captured by Magoom trap in the metallurgic region, Minas Gerais, Brazil. Ciência Rural Journal, 1997, 27, 645–649

[R32] Rodríguez-Batista B.Z., Leite R.C., Oliveira P.R., Lopes C.M.L. & Borges L.M.F. Populational dynamics of *Stomoxys calcitrans* (Linneaus) (Diptera: Muscidae) in three biocenosis, Minas Gerais, Brazil. Veterinary Parasitology, 2005, 130, 343–346 15908125 10.1016/j.vetpar.2005.03.006

[R33] Schoof H.F. Laboratory culture of *Musca*, *Fannia*, and *Stomoxys*. Bulletin of the World Health Organization, 1964, 31, 539–544 14272463 PMC2555056

[R34] Smith C.W. & Hansens E.J. The effect of temperature and humidity on the amount of blood ingested by the stable fly, *Stomoxys calcitrans* L. (Diptera: Muscidae). Journal of the New York Entomological Society,, 1975, 83, 235–240

[R35] Smith J.P., Hall R.D. & Thomas G.D. A review of natural mortality and enemies of the stable fly (Diptera: Muscidae) in Missouri. The Florida Entomologist, 1989, 72, 351–360

[R36] Sutherland B. The temperature preferences of the motile stages of *Stomoxys calcitrans* Linnaeus (Diptera: Muscidae). Onderstepoort Journal of Veterinary Research, 1980, 47, 7–11 7454236

[R37] Taylor D.B., Moon R.G. & Mark D.R. Economic impact of stable flies (Diptera: Muscidae) on dairy and beef cattle production. Journal of Medical Entomology, 2012, 49, 198–209 22308789 10.1603/me10050

[R38] Turell M.J. & Knudson G.B. Mechanical transmission of *Bacillus anthracis* by stable flies (*Stomoxys calcitrans*) and mosquitoes (*Aedes aegypti* and *Aedes taeniorhynchus*). Infection and Immunity, 1987, 55, 1859–1861 3112013 10.1128/iai.55.8.1859-1861.1987 PMC260614

[R39] Venkatesh K. & Morrison P.E. Some aspects of oogenesis in the stable fly *Stomoxys calcitrans* (Diptera: Muscidae). Journal of Insect Physiology, 1980, 26, 711–715

[R40] Venkatesh K. & Morrison P.E. Blood meal as a regulator of triacylglycerol synthesis in the haematophagous stable fly, *Stomoxys calcitrans*. Journal of Comparative Physiology, 1982, 147, 49–52

[R41] Weber A.F., Moon R.D., Sorensen D.K., Bates D.W., Meiske J.C., Brown C.A., Rohland N.L., Hooker E.C. & Strand W.O. Evaluation of the stable fly (*Stomoxys calcitrans*) as a vector of enzootic bovine leukosis. American Journal of Veterinary Research, 1988, 49, 1543–1549 2851955

[R42] Xue R., Barnard D.R. & Muller G.C. Effects of body size and nutritional regimen on survival in adult *Aedes albopictus* (Diptera: Culicidae). Journal of Medical Entomology, 2010, 47, 778–782 20939370 10.1603/me09222

[R43] Yeruham I. & Braverman Y. Skin lesions in dogs, horses and calves caused by the stable fly *Stomoxys calcitrans* (L.) (Diptera: Muscidae). Revue d’Élevage et de Médecine Vétérinaire des Pays Tropicaux, 1995, 48, 347–349 8734229

[R44] Zumpt F. The Stomoxyinae biting flies of the world. Taxonomy, biology, economic importance and control measures. Gustav Fischer Verlag, Stuttgart, Germany, 1973

